# Therapeutic Potential of Caspofungin Combined with Trimethoprim-Sulfamethoxazole for *Pneumocystis* Pneumonia: A Pilot Study in Mice

**DOI:** 10.1371/journal.pone.0070619

**Published:** 2013-08-05

**Authors:** Maria Luísa Lobo, Francisco Esteves, Bruno de Sousa, Fernando Cardoso, Melanie T. Cushion, Francisco Antunes, Olga Matos

**Affiliations:** 1 Unidade de Parasitologia Médica, Grupo de Protozoários Oportunistas/VIH e Outros Protozoários, CMDT, Instituto de Higiene e Medicina Tropical, Universidade Nova de Lisboa, Lisboa, Portugal; 2 Faculdade de Psicologia e Ciências da Educação, Universidade de Coimbra, CMDT, Coimbra, Portugal; 3 University of Cincinnati College of Medicine, Cincinnati, Ohio, United States of America; 4 Faculdade de Medicina, Hospital de Santa Maria, Universidade de Lisboa, Lisboa, Portugal; Albert Einstein College of Medicine, United States of America

## Abstract

*Pneumocystis* pneumonia (PcP) is a major cause of mortality and morbidity in immunocompromised patients. There are limited alternative therapeutic choices to trimethoprim-sulfamethoxazole (TMP-SMX) which is the standard first line therapy/prophylaxis for PcP. The efficacy of low doses of caspofungin and caspofungin in association with TMP-SMX standard-prophylactic dose was evaluated in an experimental model of *Pneumocystis*. Susceptibility of *Pneumocystis* spp. to low doses of caspofungin and caspofungin/TMP-SMX was evaluated in Balb/c immunosuppressed mice, infected intranasally with *P. murina*. Caspofungin was administered once daily at 0.1 mg/kg, 0.05 mg/kg, and 0.001 mg/kg and TMP-SMX was administered by oral gavage (12.25 mg/62.5 mg/day), for 21 days. Efficacy was calculated based on the reduction in organism burden determined through quantitative fluorescent-based real-time PCR (qPCR). Serum β-1,3-D-glucan was measured as an additional marker of infection. The present data showed that caspofungin demonstrated anti-*Pneumomocystis* effect. However, the doses administrated were too low to achieve *Pneumocystis* eradication, which suggests that echinocandin treatment should not be administrated as mono-therapy. After 21 days of treatment, *P. murina* was not detected in the lungs of mice with either TMP-SMX or caspofungin/TMP-SMX. The results showed that, even at the lowest concentrations tested, the efficacy of caspofungin in association with TMP-SMX was higher than the efficacy of either drug used alone. The administration of caspofungin/TMP-SMX was at least 1.4 times more effective against *P. murina* infection than TMP-SMX used alone. The most promising result was achieved with the combination of caspofungin 0.05 mg/kg/day with TMP-SMX 12.5 mg–62.5 mg/day, which reduced the parasite burden to undetectable levels immediately at the 14^th^ day of treatment, showing a highly marked anti-*Pneumomocystis* effect. These data suggest that the administration of low doses of caspofungin in combination with low doses of TMP-SMX may provide an improved treatment protocol for *Pneumocystis* infection clearance.

## Introduction

Invasive fungal infections are an important cause of morbidity and mortality, especially in immunocompromised and/or hospitalized patients with serious underlying diseases [1], [2]. *Pneumocystis* pneumonia (PcP) is a common and serious life-threatening opportunistic disease in hosts with impaired/debilitated immune systems, especially HIV-positive persons, but also in patients who are undergoing immunosuppressive treatments related to malignancies, connective tissue diseases or organ transplantation. These organisms are also emerging as a co-morbidity factor associated with chronic diseases such as chronic obstructive pulmonary disorder (COPD) [3]–[7].

*Pneumocystis* spp. are atypical fungi with bi-phasic life cycle in the mammalian lung, consisting of an asexual phase (binary fission of trophic forms) and a sexual cycle (conjugation of trophic forms resulting in formation of cysts). As the organism cannot be cultured reliably *in vitro*, there is a poor understanding of the life cycle, and the development of alternative therapeutic choices is limited. Therefore, drug efficacy studies have been carried out directly on rodent models [8]–[12]. PcP transmission is thought to be via airborne route. *Pneumocystis* spp. attaches to the host alveolar epithelium through Type I pneumocytes and fill the alveolar sacs. Without effective treatment, the host succumbs to respiratory failure and related organ dysfunction [6], [10].

Due to the lack of ergosterol in the cell membrane, amphotericin B and the azoles are ineffective against *Pneumocystis* spp. infections. The standard first line therapy/prophylaxis for PcP is the combination therapy, trimethoprim-sulfamethoxazole (TMP-SMX). Despite being effective, there are significant prophylactic and treatment failures as well as intolerance or side effects associated with this anti-folate inhibitors combination that may hamper the clinical outcome of the disease. Relapse and recurrence of infection are high when using secondary therapies (pentamidine, clindamycin-primaquine or atovaquone). New therapeutic approaches are needed [6], [7], [11], [13]–[15].

In the early 2000’s, echinocandins were licensed for the treatment and prevention of fungal infections. Caspofungin (caspofungin acetate) is a semi-synthetic water soluble lipopeptide (fermentation product of the fungus *Glorea lozoyensis*) and a noncompetitive inhibitor of the β(1,3)D-glucan (BG) synthase (not present in mammalian cells) that presents excellent safety profiles [1], [12], [16], [17]. This echinocandin is effective against *Pneumocystis* spp. cysts that strongly express BG synthase, but is less effective against the trophic forms, which are poor in BG. Studies suggest that treatment of PcP with caspofungin alone may not likely result in the effective eradication of the infection, which could result in relapse [3], [13], [16], [18]–[21].

There is a scarcity of data about the efficacy of the addition of caspofungin to TMP-SMX for treatment of PcP patients. A few reports suggest that the use of caspofungin with TMP-SMX regimen may lead to a faster improvement of the patient followed by complete cure of pneumonia [20], [22], however, the use of low doses of these anti-PcP molecules in association was not evaluated.

In order to improve PcP treatment and to overcome the potential intolerance and adverse effects of the standard anti-*Pneumocystis* spp. drugs, the aim of the present study was to evaluate the susceptibility of *Pneumocystis* spp. organisms to low doses of caspofungin alone and caspofungin in association with TMP-SMX, in the rodent model.

## Materials and Methods

### Animals

Specific pathogen-free BALB/c male mice were obtained from the Instituto de Higiene e Medicina Tropical, Universidade Nova de Lisboa Breeding Laboratory and were housed in filter-topped cages and fed autoclaved chow and water *ad libitum*.

### Drugs

Caspofungin (25 mg) was supplied by Merck Sharp & Dohme Corp. TMP-SMX (Bactrim®, Roche) pediatric suspension (TMP 8 mg/ml+SMX 40 mg/ml) was purchased.

### Parasites

*P. murina* (2×10^7^ nuclei/ml) for mice inoculation were purified from the lungs of C3H/HeN wild type mice. The techniques adopted have been described in earlier reports [3], [23].

### Experimental Design

**Figure 1:**
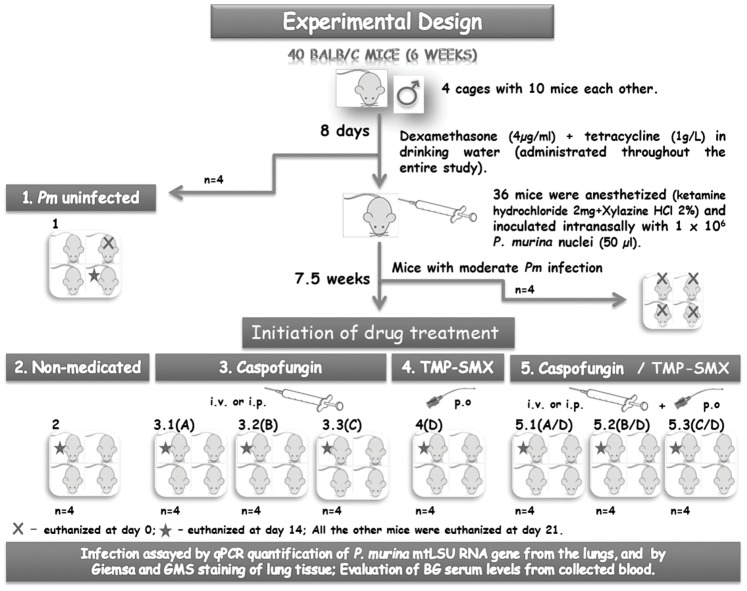
Experimental design of the study. The drug treatment was administered once-daily for 21 days. Four *P. murina*-free mice were housed separately (Group 1), serving as sentinel controls which were also sacrificed for *P. murina* detection at the time points day 0 (one mouse), day 14 (one mouse) and two mice at the end of the experiment (21^st^ day). Four mice randomly selected from the four large cages harboring immunosuppressed, *P*. *murina* infected mice, were sacrificed at the start of the study (day 0) to confirm the presence of acute *P. murina* infection. At the time points 14 and 21 days, mice were sacrificed according the following protocol: Group 2, Group 3 (subgroups 3.1, 3.2 and 3.3), Group 4 and Group 5 (subgroups 5.1, 5.2 and 5.3) - one mouse at day 14, and three mice at day 21. Legend: *Pm*- *Pneumocystis murina*; n– number of mice in cage; i.v.– intravenously injection; i.p. – intraperitoneal injection; p.o.– *per os*; A- Dose level of caspofungin: 0.1 mg/kg per day; B- Dose level of caspofungin: 0.05 mg/kg per day; C - Dose level of caspofungin: 0.001 mg/kg per day; D – TMP-SMX administered by oral gavage at 12.5 mg–62.5 mg per dose (doses of 39 µl TMP-SMX pediatric suspension were administered. The dose was calculated taking into account the average weight of 25 g per mouse and an initial suspension of TMP 8 mg/ml+SMX 40 mg/ml).

Evaluation of anti-*Pneumocystis* activity was conducted with an immunosuppressed mouse model of PcP. The experimental design of the study is adapted from Cushion *et al*., 2010 [3] and summarized in Figure 1. The immunosuppressed state was induced by administration of 4 mg/ml dexamethasone (Sigma) in the drinking water. Tetracycline (1 g/L, Sigma) was added to the water to avoid bacterial infection. Animals were anesthetized with a drug cocktail (ketamine hydrochloride 2 mg and Xylazine HCl 2%) and injected intranasally with *P. murina* at a dose of 10^6^ nuclei per mouse. Four *P. murina*-free mice were housed separately (sentinel controls, Group 1– *Pm* uninfected). When the infection reached a moderate intensity (after 7.5 weeks), mice were randomly divided into treatment and control groups of four animals each. A total of eight groups were generated (see Table 1): Group 2– Non-medicated (immunosuppressed mice without anti-*P. murina* therapy), Group 3– Caspofungin (three subgroups: 3.1, 3.2 and 3.3), Group 4– TMP-SMX (4), Group 5– Caspofungin/TMP-SMX (three subgroups: 5.1, 5.2 and 5.3). Four mice were sacrificed at the start of the study to confirm the presence of acute *P. murina* infection. Caspofungin (dissolved in 0.9% NaCl) was administered intravenously (i.v.) during the first 8 days and by intraperitoneal (i.p,) injection during the remaining 13 days of treatment on a mg/kg basis once daily for 21 days, at varying dose levels (0.1 mg/kg, 0.05 mg/Kg and 0.001 mg/Kg). The change in the treatment regimen was a consequence of the overly frequent (daily) administration of caspofungin in the mice tail vein that started to induce vein collapse, signs of necrosis in their tails and high levels of animal stress. In previous case reports of caspofungin treatment of PcP cases, standard-doses of 50–70 mg/day were usually administrated [16], [20], [21]. Also, Cushion and co-workers 2010, tested 1 mg/kg-0.1 mg/kg doses i.p. one to three times a week in the rodent model, suggesting its effectiveness at reducing cyst burdens. In the present study, lower concentrations of caspofungin were selected to test the efficacy of the concomitant use of caspofungin in association with TMP-SMX. TMP-SMX pediatric suspension was administered by oral gavage at the standard-prophylactic dose (12.5 mg–62.5 mg daily) [3], [23]. The drug treatment continued for 21 days during which time the mice remained under the immunosuppressive regimen. Control animals receiving steroids (Group 2, *P. murina* infected mice without therapy) received no treatment. At the time points 0, 14, and 21 days, mice were anesthetized and euthanized (Group 1: one mouse at day 0 and 14, respectively and two mice at day 21; four mice randomly selected from the four large cages harboring immunosuppressed *P*.*murina* infected mice prior grouping them into 8 small boxes according the distinct groups were euthanized at day 0; Group 2, Group 3– subgroups 3.1, 3.2 and 3.3, Group 4 and Group 5– subgroups 5.1, 5.2 and 5.3: one mouse at day 14, and three mice at day 21). Mice were exsanguinated, and their lungs were aseptically removed and homogenized for further histological and molecular analysis. All caging procedures and surgical manipulations were done in a laminar flow hood. The experimental protocols were approved by the “Sociedade Portuguesa de Ciências em Animais de Laboratório/Direcção Geral de Veterinária (SPCAL/DGV)”, Institutional Animal Care, in strict accordance with the recommendations in the Guide for the Care and Use of Laboratory Animals.

**Table 1:** Results of qPCR quantification of *P. murina* mtLSU RNA gene in the lungs of the nine distinct mice groups enrolled in the study.

Groups/Subgroups of mice				qPCR (average concentration)			Fold reduction (v/s untreated control)	
				Day 0	Day 14	Day 21	Day 14	Day 21
**Group 1**	***Pm*** ** uninfected control ^a^**			MB	MB	MB	–	–
**Group 2**	***Pm*** ** infected, untreated control^b,c^**			7.17×10^8^	7.40×10^8^	7.94×10^8^	–	–
**Group 3**	***Pm*** ** infected, Caspofungin^b,c^**	**3.1**	(0.1 mg/kg/day)	7.17×10^8^	3.55×10^8^	2.59×10^8^	2.1	3.1
		**3.2**	(0.05 mg/kg/day)	7.17×10^8^	1.93×10^8^	3.93×10^8^	3.8	2.0
		**3.3**	(0.001 mg/kg/day)	7.17×10^8^	2.02×10^9^	6.46×10^8^	–	1.2
**Group 4**	***Pm*** ** infected, TMP-SMX^b,c^**	**4**	(12.25 mg–62.5 mg/day)	7.17×10^8^	1.13×10^7^	MB	65.5	UD
**Group 5**	***Pm*** ** infected, Caspofungin/TMP-SMX^b,c^**	**5.1**	(0.1 mg/kg/day+12.5 mg–62.5 mg/day)	7.17×10^8^	5.67×10^6^	MB	130.5	UD
		**5.2**	(0.05 mg/kg/day+12.5 mg–62.5 mg/day)	7.17×10^8^	MB	MB	ND	UD
		**5.3**	(0.001 mg/kg/day+12.5 mg–62.5 mg/day)	7.17×10^8^	7.98×10^6^	MB	92.7	UD

**Note:** The level of infection of the samples was estimated using the standard curve determined in the study: Cq = −5.3661 log_10_ (mtLSU rRNA concentration) +68.7078; R^2^  = 0.9933; Quantification cycles (Cq) were obtained in duplicate and the average Cq were calculated. The maximum Cq was 42.82 (dilution 1∶300 of the initial *P. murina* 2×10^7^ nuclei/ml suspension). *Pm- Pneumocystis murina;* ^a^The control mice which had not been infected by *P. murina*, did not presente *Pneumocystis* DNA in the lungs, indicating that the animals used in the study were free of *P. murina* prior to exposure to the microorganism. The Group 1 qPCR values is an average of one mouse dosed in duplicate for days 0 and day 14, respectively and two mice dosed each in duplicate for day 21; ^b^ The same fungal burden at day 0 resulted from the average of four mice each dosed in duplicate; ^c^ the qPCR values is an average of one mouse dosed in duplicate for day 14, and three mice dosed each in duplicate for day 21; MB – below the minimal fungal burden detectable by qPCR; UD - reduction to undectable levels.

### Evaluation of *P. murina* Infection Levels

Extraction of DNA from mice lungs was performed using a Mini-BeadBeater/guanidinium thiocyanate-silica method, as described previously [24], [25]. Infection levels were determined by a quantitative fluorescence-based real-time PCR (qPCR) for *P. murina* mitochondrial large-subunit rRNA gene (mtLSU rRNA) quantification. The qPCR assays (Taqman® MGB probes, FAM™ dye-labelled, Applied Biosystems) were performed in the 7300 Real-Time PCR System (Applied Biosystems, Foster City, CA), based on data reported previously [4], [26]. The baseline was taken from cycles three to 20 and the threshold was set at 0.3. The maximum Cq value was determined at dilution 1∶300 from the initial *P. murina* 2×10^7^ nuclei/ml suspension. To convert the threshold cycle data into *P. murina* nuclei, a standard curve was generated using DNA isolated from a purified sample with 10^7^ *P. murina* nuclei/ml.

### Microscopic Evaluation of *P. murina* Infection

Smears from the homogenized lungs were stained with Giemsa and Grocott’s methenamine silver (GMS) to confirm the presence of *P. murina* organims. Slides were examined in a blinded manner using an Olympus BX51 microscope [3], [27].

### Serum β(1,3)D-glucan Content

The BG serum levels from mice were measured using the FUNGITELL™ assay (Associates of Cape Cod, Inc.,East Falmouth, MA) and performed in the NanoQuant Infinite M200 Pro (Tecan) [28].

### Data Analysis

The efficacy of the anti-*Pneunocystis* therapy regimens was evaluated calculating the decrease of organism burden by qPCR in treated mice in comparison with untreated mice (fold reduction). Statistical tests were applied to investigate associations at a significance level of 0.05 using SPSS v.20.0 software (SPSS Inc., Chicago, IL, USA). The non-parametric Kruskal-Wallis test was used to investigate differences between the median *P. murina* burden distribution variations across the groups of mice treated with different anti-*Pneumocystis* regimens.

## Results

### Evaluation of *P. murina* Infection Levels

The results obtained for qPCR quantification of *P. murina mtLSU RNA* gene in the nine distinct mice groups enrolled in this study are summarized in Table 1. Mice from negative control group remained uninfected during the time of the experiment. The *P. murina* infected untreated control had a 1.1-fold increase of organisms per lung during the 21 days. The caspofungin treatment lowered the average organisms count per lung: in subgroup 3.1 (0.1 mg/Kg/day) a 2.1-fold reduction (day 14), and a 3.1-fold reduction at day 21; in subgroup 3.2 (0.05 mg/Kg/day) a 3.8-fold reduction (day 14) and a 2.0-fold reduction (day 21); in subgroup 3.3 (0.001 mg/Kg/day) was observed an initial increase of 272-fold (day 14) and a reduction of 1.2-fold (day 21) during the 21 days of the experiment. The TMP-SMX treatment decreased 65.5-fold the average of organisms count per lung in Group 4 (12.5 mg–62.5 mg/day) at day 14 and to undetectable levels at the end of the study (day 21).

The addition of low doses of caspofungin to TMP-SMX enhanced the efficacy of the treatment, dropping the average organisms count per lung (day 14): in subgroup 5.1 (0.1 mg/Kg/day caspofungin; 12.5 mg–62.5 mg/day TMP-SMX) a 130.5-fold reduction; in subgroup 5.2 (0.05 mg/Kg/day caspofungin; 12.5 mg–62.5 mg/day TMP-SMX) a marked reduction, from 7.17×10^8^ to undetectable levels; and in subgroup 5.3 (0.001 mg/Kg/day caspofungin; 12.5 mg–62.5 mg/day TMP-SMX) a 92.7-fold reduction. All caspofungin/TMP-SMX regimens exhibited very effective anti-*P. murina* activity, reducing organisms counts to undetectable levels at the 21^st^ day.

Therefore, at the end of the 21^st^ day, both regimens TMP-SMX alone and Caspofungin in association with TMP-SMX reduced *P. murina* counts to undetectable levels. However, at the 14^th^ day, the combination caspofungin/TMP-SMX demonstrated higher effectiveness against *P. murina* infection than TMP-SMX alone. In comparison with the TMP-SMX regimen, the combination 0.05 mg/Kg/day caspofungin with 12.5 mg–62.5 mg/day TMP-SMX was more effective, reducing the average *P. murina* burden to undetectable levels immediately at day 14. Also, the concentrations 0.01 mg/Kg/day caspofungin with 12.5 mg–62.5 mg/day TMP-SMX and 0.001 mg/Kg/day caspofungin with 12.5 mg–62.5 mg/day TMP-SMX suggested a 2.0 times and a 1.4 times more effective fold reduction than TMP-SMX alone, respectively.

### Microscopic Evaluation of *P. murina* Infection

The presence of *P. murina* organisms was confirmed in the lung smears of infected animals by the staining procedures adopted. Samples with undetectable levels by qPCR showed no *P. murina* organisms. As expected, no *P. murina* organisms were observed in the lungs of the sentinel control mice. All samples were analyzed using this quality control.

### Serum β(1,3)D-glucan Content

The levels of BG measured in the serum of the rodents, at days 0 and 21, are presented in Table 2. As expected, the decrease of *P. murina* organisms in all the treated mice correlated with a reduction of BG content. However, the reduction in BG levels was not proportional to the decrease of *P. murina* organisms. At day 21, the lowest levels of BG (68.94 pg/ml) were observed in mice treated with 0.05 mg/Kg/day caspofungin, followed by the TMP-SMX regimen (75.53 pg/ml). With the exception of group 5.2 (0.05 mg/Kg/day caspofungin; 12.5 mg–62.5 mg/day TMP-SMX), the BG content in the mice treated with the combination caspofungin/TMP-SMX was lower than in the ones treated with caspofungin in mono-therapy.

**Table 2:** Results of serum BG levels from mice enrolled in the study.

Groups/subgroups of mice				β-1,3-D-glucan (pg/ml)	
				0 day	21 day
**Group 1**	***Pm*** ** uninfected control**			73.93	76.72
**Group 2**	***Pm*** ** infected, untreated control**			470.36	310.23
**Group 3**	***Pm*** ** infected, Caspofungin**	**3.1**	(1 mg/kg/day)	470.36	301.57
		**3.2**	(0.05 mg/kg/day)	470.36	68.94
		**3.3**	(0.001 mg/kg/day)	470.36	216.34
**Group 4**	***Pm*** ** infected, TMP-SMX**	**4**	(12.25 mg–62.5 mg/day)	470.36	75.53
**Group 5**	***Pm*** ** infected, Caspofungin/TMP-SMX**	**5.1**	(0.1 mg/kg/day+12.5 mg–62.5 mg/day)	470.36	197.92
		**5.2**	(0.05 mg/kg/day+12.5 mg–62.5 mg/day)	470.36	214.36
		**5.3**	(0.001 mg/kg/day+12.5 mg–62.5 mg/day)	470.36	104.35

**Note:** The reference values established for fungal infections and the baseline limits of the method are optimized to measure the β-1,3-D-glucan serum/plasma levels from humans. However the same values were adopted for this study: Negative: <60 pg/ml; Indeterminate: 60–79 pg/ml; Positive: >80 pg/ml; Upper limit: ≤500 pg/ml; Lower limit ≥31 pg/ml.

### Data Analysis

The Kruskal-Wallis test showed no statistical significances between the median *P. murina* loads distribution variation across the distinct groups (TMP-SMX or caspofungin/TMP-SMX treatment regimens) studied by qPCRs, mainly due to the small number of mice in each combination of treatment and doses. Nevertheless, if mice are grouped according to treatment regimen, regardless of the concentrations of drug administered (group 2 - *P. murina* infected untreated mice; group 3 - *P. murina* infected mice treated with caspofungin; group 4 - *P. murina* infected mice treated with TMP-SMX; group 5 - *P. murina* infected mice treated with caspofungin/TMP-SMX), there are some relevant statistical associations. Kruskal-Wallis test rejected the null hypothesis that the median *P. murina* burden are the same in treated (caspofungin, TMP-SMX, or caspofungin/TMP-SMX) and untreated mice, rejecting the equality of the median values at the 21^st^ day between group 5 and group 2 (caspofungin/TMP-SMX *versus* untreated, *P*<0.001; Std. error 4.877), group 4 and group 2 (TMP-SMX *versus* untreated, *P*  = 0.016; Std. error 6.678) and group 5 and group 3 (caspofungin/TMP-SMX *versus* TMP-SMX, *P*<0.023; Std. error 4.702).

## Discussion

There is a lack of clinical experience with using caspofungin to treat PcP. Nevertheless, few studies raised the importance of the potential clinical use of this BG synthase inhibitor [1], [13], [20]–[22]. In the present study *Pneumocystis* spp. susceptibility to low doses of caspofungin and caspofungin in association with TMP-SMX, in the rodent model was evaluated. The obtained data demonstrated that addition of caspofungin (active against cysts) in low doses to TMP-SMX (active against trophic forms and cysts) in prophylactic doses may provide an additive anti-*Pneumomocystis* effect, completely inhibiting *Pneumocystis* spp. life cycle. Our results suggest that the administration of low doses of caspofungin in combination with low doses of TMP-SMX provides an improved hastening clearance of the infection, as they are faster acting over *Pneumocystis* spp. organisms than either TMP-SMX alone, or caspofungin in mono-therapy.

In the course of the 21 days of PcP treatment with caspofungin mono-therapy, the highest concentration (0.1 mg/Kg/day) tested showed the most effective anti-*Pneumocystis* activity, followed by the second highest concentration (0.05 mg/Kg/day) and by the lowest concentration (0.001 mg/Kg/day) (Table 1), as expected. Nevertheless, on the 21^st^ day, *P. murina* was still detectable in the lungs of mice, adding evidence that the doses administrated were too low to achieve an effective treatment, or that this drug alone was unable to eradicate *Pneumocystis* spp. infection. These results confirmed the evidence that echinocandin treatment should not be administrated as mono-therapy. Since these compounds chiefly target the cysts and are less effective against the trophic forms, it should be taken into account that cessation of echinocandin treatment while the host remains immunosuppressed could result in inadvertent cyst repopulation and subsequent relapse as reported previously [3], [12], [16], [20]. Unlike the other groups under treatment, in which effective decreases of organisms were detected in days 14 and 21, it was observed that in the group of mice treated with 0.001 mg/Kg/day caspofungin there was an initial 272-fold (day 14) increase of *P. murina* organisms, followed by a 1.2-fold (day 21) reduction. These results may be a consequence of the particularly low dose of caspofungin administrated in this group, which probably has a weaker anti-*P. murina* effect, delaying the clearance of infection. A higher fungal load was observed at 14^th^ day in mice treated with caspofungin (0.001 mg/Kg/day) than in untreated controls. We hypothesize that such changes may be associated to intrinsic features of each animal, namely to mice inter-individual dissimilarities, reported in rodent models by other authors [29].

The present data showed that the concomitant use of caspofungin in low doses and TMP-SMX in lower doses was effective against *Pneumocystis* spp. infection, reducing the parasite burden to undetectable levels in the lungs of mice at the end of the 21 days of treatment. The qPCR data clearly showed that even at the lowest concentrations administrated (caspofungin 0.001 mg/Kg/day; TMP-SMX 12.5 mg - 62.5 mg/day), the efficacy of the association was higher than the one observed for the drugs used alone, whatever the concentrations tested (Table 1). The most interesting and promising result was achieved by using the combination of caspofungin 0.05 mg/kg/day with TMP-SMX 12.5 mg–62.5 mg/day, which reduced the parasite burden to undetectable levels immediately at the 14^th^ day of treatment. Although additional studies are needed to confirm the trend observed the results evidenced that the use of caspofungin in association with TMP-SMX may provide an additive effect against *Pneumocystis* spp. organisms [3], [20], [22], even when low concentrations are administrated. Taking into account the concentrations used, the concomitant administration of caspofungin in association with TMP-SMX was at least 1.4 times more effective against *P. murina* infection than TMP-SMX mono-therapy, suggesting a highly marked anti-*Pneumomocystis* effect.

The lack of statistical significance of the difference between the median *P. murina* burden distribution variation across the distinct groups resulted from the small number of mice enrolled in each group (four mice), making it meaningless to test the assumptions of normality and homogeneity of variance necessary to apply the t-test. In order to overcome this limitation, the results of the median *P. murina* burden were pooled according to the treatment regimens. Taking into consideration the significance levels, this approach strongly supports the hypotheses that at the end of the treatment: 1) caspofungin/TMP-SMX combination was statistically more effective than caspofungin in mono-therapy, 2) both TMP-SMX and caspofungin/TMP-SMX were statistically effective treatments to deplete *P. murina* infection, in the rodent model. The treatment with caspofungin/TMP-SMX showed the highest significance level (*P*<0.001), which suggests this therapeutic association as a potential candidate regimen to treat PcP that should be studied in further investigations.

The potential additive effect of caspofungin/TMP-SMX combined therapy against *Pneumocystis* spp. infection may be advantageous in the treatment of PcP patients, as it appears to fully inhibit the organism’s life cycle and promote a faster recovery and a better outcome when compared with the therapy standards normally applied. The use of these molecules in combination produces a double effect: (1) fungistatic effect that results from (a) the blockade of the cell wall synthesis by caspofungin, and (b) nucleic acids and protein breakdown by TMP-SMX, reducing the trophic forms and consequently the cysts formation; (2) fungicidal effect that results from (a) a change in the integrity of the cell wall, which loses its mechanical strength and becomes unable to resist the intracellular osmotic pressure through the action of caspofungin, and from (b) the action of TMP-SMX that impairs the function of folic acids, promotes an inability to translate and synthesize proteins, which conducts a metabolic knock-out, both leading ultimately to destruction of the *Pneumocystis* spp. cells [3], [15], [16], [30]. Additionally, the use of caspofungin in combination with TMP-SMX may decrease the harsh inflammatory responses linked to BG, a well-known pro-inflammatory factor implicated in detrimental inflammatory responses in PcP, as reported previously [3], [10], [31]–[34].

As expected the BG serum levels decreased throughout the treatment period for all the drugs tested. Although the reduction in BG levels was not proportional to the decrease in *P. murina* organisms, the present data confirmed the efficacy of the compounds used. 0.05 mg/Kg/day caspofungin mono-therapy and 12.5 mg–62.5 mg/day TMP-SMX have shown the highest BG serum levels decreases. Overall, the BG results suggest that the combination caspofungin/TMP-SMX was more efficient than caspofungin in mono-therapy to treat PcP. This data may be related to the fact that: a) TMP-SMX targets trophic forms resulting mostly in anti-*Pneumomocystis* activity without fluctuations in the BG basal levels; b) caspofungin destroys the cystic forms of *Pneumocystis* followed by elevation of serum BG. Additionally, other authors proposed the possibility that immunocompromised hosts clear BG slowly, remaining longer in the bloodstream [3], [33].

In conclusion, the results of this study suggest but do not prove the potential benefit of the combined PcP treatment caspofungin/TMP-SMX in lower doses in comparison with those tested in previous studies [3]. Further studies involving a larger number of mice and time points over the treatment period are required, to confirm the efficacy of this drug combination and to determine the shortest period of drugs administration. This new approach provided a significant contribution to the development and enrichment of anti-*Pneumomocystis* armamentarium, contributing for further selection of drugs that lead to a reliably, minimally toxic, better tolerated, highly effective and time/cost saving treatment for PcP.
